# Exercise cardiovascular magnetic resonance shows improved diastolic filling by atrioventricular area difference in athletes and controls

**DOI:** 10.1152/japplphysiol.00446.2024

**Published:** 2024-10-17

**Authors:** Jonathan Edlund, Björn Östenson, Einar Heiberg, Håkan Arheden, Katarina Steding-Ehrenborg

**Affiliations:** Clinical Physiology, Department of Clinical Sciences Lund, Lund University, Lund, Sweden; Department of Clinical Physiology, Skåne University Hospital, Lund, Sweden

**Keywords:** atrioventricular area difference, exercise cardiac magnetic resonance imaging, hydraulic force, left atrium, left ventricle

## Abstract

Hydraulic force, a novel mechanism shown to aid diastolic filling, can be calculated by assessing the geometrical relationship between the left ventricular and atrial short-axis areas (atrioventricular area difference, AVAD) (Maksuti E, Carlsson M, Arheden H, Kovács SJ, Broomé M, Ugander M. *Sci Rep* 7: 43505–43510, 2017; Steding-Ehrenborg K, Hedström E, Carlsson M, Maksuti E, Broomé M, Ugander M, Magnusson M, Smith JG, Arheden H. *J Appl Physiol (1985)* 130: 993–1000, 2021). During exercise both ventricular and atrial volumes change due to altered loading conditions compared with rest, but it is unknown to what extent this affects AVAD. The aim of this study was to investigate whether AVAD differs when going from rest to exercise in sedentary controls and athletes. We included 13 sedentary controls and 20 endurance athletes to undergo cardiovascular magnetic resonance (CMR) imaging at rest and during moderate and vigorous exercise using a CMR-compatible ergometer. AVAD was calculated as the largest ventricular short-axis area minus the largest atrial short-axis area in end-diastole (ED) and end-systole (ES) as measured from CMR short-axis images. AVAD in ED increased during moderate exercise in both sedentary controls and athletes, thus aiding diastolic filling, but did not increase further during vigorous exercise. AVAD in ES was negative in both groups at rest and decreased further with increasing exercise intensity in sedentary controls, whereas athletes remained unchanged. In conclusion, results from AVAD in ED indicate the net hydraulic force to further augment diastolic filling during moderate exercise when compared with rest, providing new insights into the mechanism by which diastolic function increases during exercise.

**NEW & NOTEWORTHY** This study is the first to assess hydraulic force during exercise, a novel mechanism shown to augment diastolic filling at rest. Our results indicate hydraulic force to further aid in diastolic filling during moderate exercise compared with rest in athletes and sedentary controls, providing new insights into the mechanism by which the left ventricle increases diastolic function during exercise.

## Graphical abstract

**Figure:**
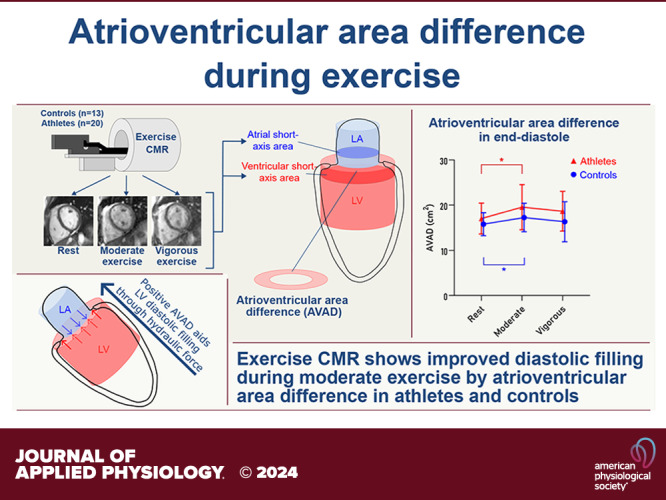

## INTRODUCTION

Endurance athletes are known to be able to increase their cardiac output to a larger extent than untrained individuals (1). Furthermore, endurance athletes can maintain or even augment the stroke volume during strenuous exercise, whereas studies indicate untrained individuals to plateau or even decrease in stroke volume as they approach maximum heart rate (2–5). The mechanisms enabling athletes to maintain stroke volume when approaching maximum heart rate are not yet fully understood. However, studies indicate endurance athletes have increased diastolic function compared with controls, when measured at rest (6–8). We hypothesize that there may be differences comparing rest to exercise and during exercise between athletes and sedentary controls in diastolic filling, measured as hydraulic force.

**Figure 1.**
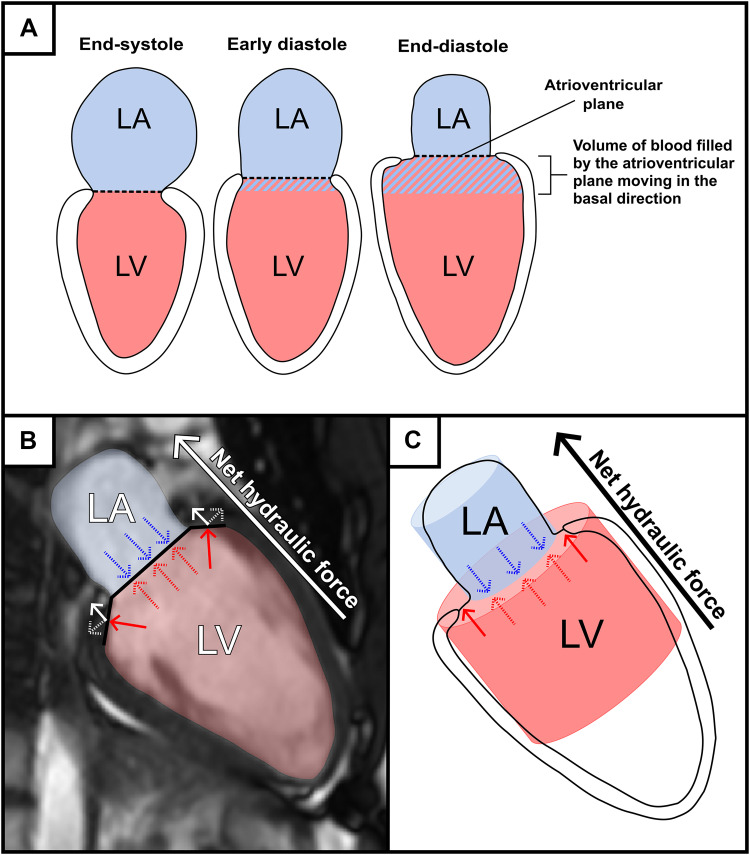
Visualizations of net hydraulic force augmenting left ventricular diastolic filling in a healthy heart by applying force to the atrioventricular plane in the basal direction. *A*: stylized figures of the left atrium (LA, shaded in blue) and ventricle (LV, shaded in red), divided by the atrioventricular plane (dashed line) in end-systole, early diastole, and end-diastole. The blue-striped area represents the volume of blood which is filled by displacing the atrioventricular plane throughout diastole in the basal direction. *B*: a cardiovascular magnetic resonance two-chamber view of the left atrium (shaded in blue) and ventricle (shaded in red), separated by the atrioventricular plane (black line). Hydraulic force is exerted by the blood on the atrioventricular plane both from the atrium (blue arrows) and the ventricle (red arrows). The hydraulic force cancels out where the areas of the two chambers overlap (dashed red and blue arrows). The hydraulic force exerted where the chambers do not overlap (solid red arrows) can be divided into radial (dashed white arrow) and longitudinal (white arrow) components of the hydraulic force. The radial component is counteracted by the stiff pericardium, while the longitudinal component contributes to pushing the atrioventricular plane in the basal direction. Thus, the net hydraulic force will be in the basal direction, augmenting ventricular filling by displacing the atrioventricular plane. This visualization is simplified in (*C*), with a stylized two-chamber view and the two chambers represented as two cylinders. Here it becomes more apparent that a larger short-axis area leads to a larger hydraulic force, which is proportional to the difference in surface area between the two cylinders. This is the atrioventricular area difference.

The hydraulic force in the heart is a novel mechanism that has been shown to aid diastolic filling in healthy volunteers and athletes, and to impair diastolic filling in patients with heart failure with preserved ejection fraction and after heart transplantation (9–11). Hydraulic force can be described as the pressure a fluid exerts on a given area (force = pressure × area). In the setting of the left side of the heart, the fluid generating the force is the blood, and the area it exerts on is the endocardium of both the atrium and ventricle. During diastole, the mitral valve is open and the pressure difference between the atrium and ventricle is close to zero, and measurements of pressure can therefore be omitted. The direction of the net hydraulic force can thus be assessed by calculating the cross-sectional area difference between the left ventricle (LV) and left atrium (LA), termed the atrioventricular area difference (AVAD) (Fig. 1). When the atrial area is smaller than the ventricular area, the net hydraulic force aids diastolic ventricular filling (9).

The aim of this study was to investigate whether AVAD changes when going from rest to moderate and vigorous exercise in sedentary controls and athletes, using exercise cardiovascular magnetic resonance (CMR).

## MATERIALS AND METHODS

This study was approved by the regional ethics committee in Lund, Sweden (Dnr 741/2004 with complementary Dnr 269/2005) and follows the Declaration of Helsinki. Written informed consent was obtained from all participants. This study was designed as a single-center prospective cross-sectional observational study and complies with the STROBE guidelines for observational studies (12).

### Study Population

We recruited 20 athletes and 13 sedentary controls through contact with a local triathlon association, by digital and physical advertisements, and by asking friends, colleagues, and family. Sedentary controls were age- and sex-matched to the athletes, had no history of cardiovascular disease at the time of inclusion and exercised less than 150 min of moderate intensity aerobic exercise per week. Study inclusion spanned from 2017 to 2019.

### Cardiovascular Magnetic Resonance Imaging

All CMR examinations were performed using a 1.5 T scanner (Siemens Aera, Siemens Healthineers, Erlangen, Germany). For imaging at rest, we used standard ECG-gated balanced steady-state free precession (SSFP) sequences during breath-hold. Typical image parameters were: echo time 1.1 ms, repetition time 2.2 ms, flip angle 67°, acquired in-plane spatial resolution 1.9 × 1.9 mm^2^, slice thickness 8 mm with no slice gap, and reconstructed temporal resolution 40 ms.

For imaging during exercise, we used a Siemens product real-time SSFP sequence in free breathing and without ECG-triggering. Typical imaging parameters for the real-time sequence were: echo time 1.1 ms, repetition time 2.2 ms, flip angle 60°, acquired in-plane spatial resolution 1.9 × 2.8 mm, slice thickness 10 mm with no slice gap. Parallel imaging (GRAPPA factor 3) and partial Fourier (factor 5/8) acceleration were used to enable an acquired temporal resolution of 28–37 ms.

We acquired short-axis images with slices covering both ventricles and atria, and long-axis images in two-chamber, three-chamber, and four-chamber view for the purposes of this study. A single slice was acquired at rest per long-axis image, and three slices during exercise. All resting images were acquired prior to exercise.

### Exercise Protocol

Research participants performed exercise while in supine position in the scanner using an MR-compatible cycle ergometer (Lode, Groningen, The Netherlands). Exercise started at a resistance of 50 W and was ramped up manually by the operator until desired heart rate was reached. The desired intensity was individually set for each participant based on the age-predicted maximum heart rate using the formula (220 − age in years) beats per minute. Image acquisition commenced during on-going exercise at a fixed resistance once the desired heart rate [60% for moderate, 80% for vigorous (13)] was reached.

### Cardiopulmonary Exercise Testing

All participants underwent cardiopulmonary exercise testing (CPET) within an average of 6 days (range 2 to 21 days) of the CMR exam. CPET exams were performed according to clinical routine and peak oxygen consumption (V̇o_2 _peak) was used to assess overall cardiopulmonary fitness.

### Data Analysis

All image analysis was performed in Segment version 4.0 R11877 (14). Exercise CMR images were analyzed using a real-time analysis tool for respiratory compensation as previously described (15). *Observer 1* (JE, 5 yr of CMR experience) performed delineations of the LA and *observer 2* (BÖ, 6 yr of CMR experience) the LV.

### Cardiac Parameters

Cardiac parameters were derived from the manual delineations of short-axis CMR images in all slices covering the LV and LA in ventricular end-diastole (ED) and end-systole (ES). One short-axis image stack was used for analysis per exercise intensity (rest, moderate exercise, and vigorous exercise) for each participant. Cardiac parameters included heart rate, cardiac output, LV end-diastolic volumes (EDV) and end-systolic volumes (ESV), LA volumes at ventricular ED and ES, AVAD at ventricular ED and ES, and LV and LA maximum short-axis areas at ventricular ED and ES. We calculated heart rate during exercise by manually counting cardiac cycles in the acquired real-time short-axis images.

### Atrioventricular Area Difference

The hydraulic forces in the heart come from the blood exerting a force on the myocardium. This force is always present and is aimed in all directions. In physics, a force can be calculated as pressure times area. During diastole, when the mitral valve is open, the pressure difference between the LA and LV can be assumed small enough to be considered negligible. Thus, the direction of the net hydraulic force can be determined by the short-axis area difference between the atrium and ventricle, AVAD. However, the magnitude of the hydraulic force cannot be quantified without the addition of measuring invasive pressures.

**Figure 2.**
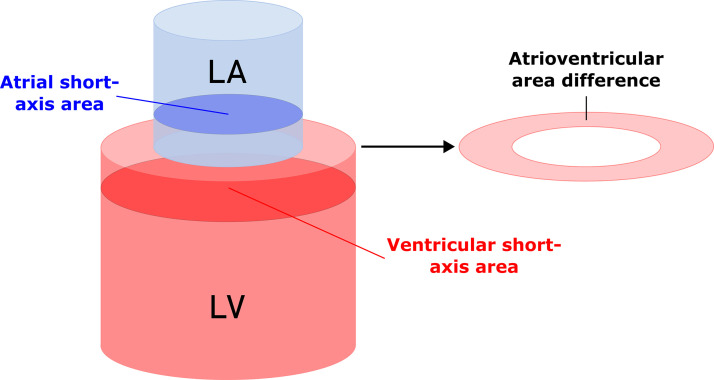
Schematic overview visualizing the atrioventricular area difference (AVAD). The atrium and ventricle are represented as blue (atrium) and red (ventricle) cylinders stacked on top of each other. The atrioventricular area difference is calculated by taking the ventricular short-axis area and subtracting the atrial short-axis area. LA, left atrium; LV, left ventricle.

Atrioventricular area difference was calculated as previously described by Maksuti et al. (9), visualized in Fig. 2. In brief, the largest endocardial ventricular and atrial short-axis areas in ventricular ED and ES were identified. Thereafter, AVAD was calculated for both ventricular ED and ES using the following equation:

$$\text{Atrioventricular area difference }\left({\text{cm}}^{2}\right)=\mathrm{ventricular short}\text{-}\mathrm{axis area}\left({\text{cm}}^{2}\right)-\mathrm{atrial short}\text{-}\mathrm{axis area}\left({\text{cm}}^{\text{2}}\right)$$

This process was repeated in each short-axis image stack for all exercise intensities for each participant, providing AVAD in ED and ES for rest, moderate exercise, and vigorous exercise in sedentary controls and endurance athletes.

### Intra- and Interobserver

Ten datasets (two rest, eight exercise) randomly chosen from the entire data set were used for assessing both intra- and interobserver variability. Intraobserver variability was assessed with at least 6 mo between delineating the datasets. Interobserver variability was tested against delineations done by *observer 3* (KSE, >15 yr of CMR experience).

### Statistical Analysis

Statistical analysis was performed in SPSS 27 & GraphPad 9.5.1. Two-way repeated-measures ANOVA with Bonferroni correction was used to test for between-group differences and interaction in AVAD and LV and LA short-axis areas. Repeated-measures ANOVA with Tukey’s multiple comparisons test was used to further test for within-group differences in AVAD and short-axis areas between rest, moderate, and vigorous exercise. Unpaired Student’s *t* test was used to compare resting cardiac parameters and participant characteristics between groups. Bland–Altman plots were used to evaluate inter- and intraobserver variability. Values are presented as means ± standard deviation.

## RESULTS

**Figure 3.**
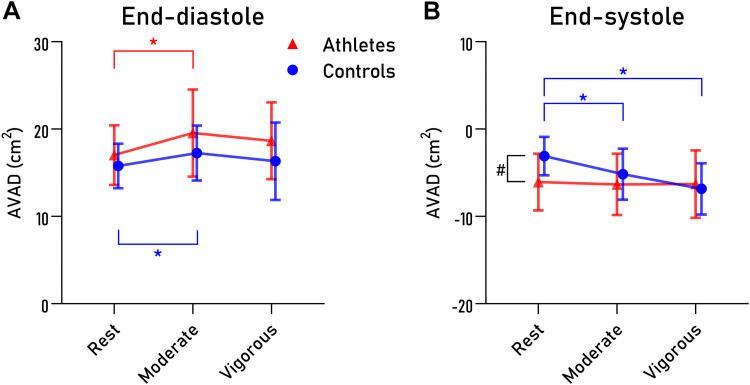
Atrioventricular area difference (AVAD) in ventricular end-diastole (*A*) and end-systole (*B*) at different exercise intensities. Endurance athletes are denoted as red triangles, sedentary controls as blue circles. Symbols and bars denote mean and standard deviation, respectively. *P < 0.05 within groups, #*P* < 0.05 between groups.

**Figure 4.**
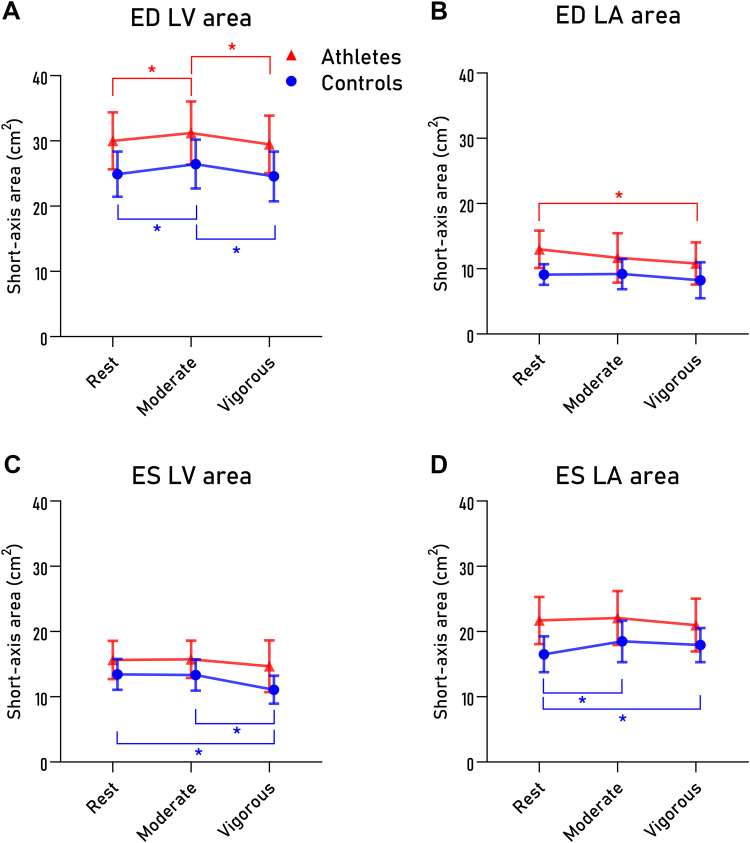
Left ventricular (LV) and left atrial (LA) maximum short-axis area measured in ventricular end-diastole (ED), shown in *A* and *B*, and in ventricular end-systole (ES), *C* and *D*, at different exercise intensities. Endurance athletes are denoted as red triangles, sedentary controls as blue circles. Symbols and bars denote mean and standard deviation, respectively. *P < 0.05 within groups.

Twenty endurance athletes and 13 sedentary controls were included. Participant characteristics are presented in Table 1. Cardiac parameters at rest, moderate, and vigorous exercise are presented in Table 2. Values for AVAD are presented in Table 3 and Fig. 3. LV and LA short-axis areas are presented in Table 3 and Fig. 4.

**Table 1.** Research participant characteristics at rest

	Sedentary Controls (*n* = 13)	Athletes (*n* = 20)	*P* Value
Age, yr	40 ± 12	40 ± 10	ns
Female:male ratio (*n*)	6:7	9:11	ns
Height, cm	176 ± 5	172 ± 8	ns
Weight, kg	69 ± 7	67 ± 9	ns
BSA, m^2^	1.83 ± 0.10	1.78 ± 0.15	ns
Systolic blood pressure, mmHg	118 ± 16	123 ± 13	ns
Diastolic blood pressure, mmHg	73 ± 10	75 ± 8	ns
Heart rate, beats/min	60 ± 6	50 ± 7	<0.001
Peak oxygen consumption, mL/kg/min	38.4 ± 7.1	53.5 ± 5.9	<0.001
LV EDV, mL	170 ± 31	215 ± 39	<0.001
LV EDV, indexed, mL/m^2^	92 ± 14	120 ± 16	<0.001
LV ESV, mL	69 ± 18	88 ± 21	0.012
LV ESV, indexed, mL/m^2^	38 ± 9	49 ± 9	0.002
LV stroke volume, mL	101 ± 14	127 ± 22	<0.001
LV stroke volume index, mL/m^2^	55 ± 6	71 ± 10	<0.001
LV ejection fraction, %	60 ± 4	59 ± 4	ns
Cardiac output, L/min	6.03 ± 0.90	6.33 ± 1.28	ns
Cardiac index, L/min/m^2^	3.29 ± 0.47	3.55 ± 0.65	ns
LA volume at ventricular ES, mL	76 ± 15	102 ± 21	<0.001
LA volume at ventricular ES, indexed, mL/m^2^	41 ± 7	57 ± 10	<0.001
LA volume at ventricular ED, mL	29 ± 7	46 ± 13	<0.001
LA volume at ventricular ED, indexed, mL/m^2^	16 ± 3	25 ± 7	<0.001

Values are means ± SD. BSA, body surface area; EDV, end-diastolic volume; ESV, end-systolic volume; LA, left atrial; LV, left ventricular. ns *P* > 0.05.

**Table 2.** Cardiac parameters at rest, moderate and vigorous exercise

Sedentary Controls (*n* = 13)	Rest	Moderate Exercise	Vigorous Exercise
Heart rate, beats/min	60 ± 6	122 ± 12	151 ± 10
Cardiac output, L/min	6.0 ± 0.9	13.2 ± 2.5	15.3 ± 4.3
LV EDV, mL	170 ± 31	176 ± 34	155 ± 32
LV ESV, mL	69 ± 19	67 ± 19	55 ± 14
LA volume at ventricular ES, mL	76 ± 15	92 ± 20	85 ± 19
LA volume at ventricular ED, mL	30 ± 7	31 ± 9	29 ± 11

Athletes (*n* = 20)	Rest	Moderate Exercise	Vigorous Exercise
Heart rate, beats/min	50 ± 7	128 ± 14	152 ± 17
Cardiac output, L/min	6.3 ± 1.3	17.6 ± 3.6	19.9 ± 2.7
LV EDV, mL	215 ± 39	215 ± 37	199 ± 30
LV ESV, mL	88 ± 21	79 ± 21	67 ± 19
LA volume at ventricular ES, mL	102 ± 21	111 ± 27	102 ± 24
LA volume at ventricular ED, mL	46 ± 13	43 ± 17	36 ± 13

Values are means ± SD. ED, end-diastole; EDV, end-diastolic volume; ES, end-systole; ESV, end-systolic volume; LA, left atrial; LV, left ventricular.

**Table 3.** Short-axis areas and atrioventricular area difference at rest, moderate and vigorous exercise

Sedentary Controls (*n* = 13)	Rest	Moderate Exercise	Vigorous Exercise
LV short-axis area, ventricular ED, cm^2^	24.9 ± 3.5	26.5 ± 3.8*	24.6 ± 3.8†
LA short-axis area, ventricular ED, cm^2^	9.1 ± 1.6	9.2 ± 2.3	8.2 ± 2.8
AVAD, ventricular ED, cm^2^	15.8 ± 2.6	17.3 ± 3.1*	16.3 ± 4.4
LV short-axis area, ventricular ES, cm^2^	13.4 ± 2.4	13.3 ± 2.4	11.1 ± 2. 1#†
LA short-axis area, ventricular ES, cm^2^	16.5 ± 2.7	18.5 ± 3.2*	17.9 ± 2.6#
AVAD, ventricular ES, cm^2^	−3.1 ± 2.2	−5.2 ± 2.9*	−6.8 ± 2.9#

Athletes (*n* = 20)	Rest	Moderate Exercise	Vigorous Exercise
LV short-axis area, ventricular ED, cm^2^	30.0 ± 4.4	31.2 ± 4.8*	29.5 ± 4.4†
LA short-axis area, ventricular ED, cm^2^	13.0 ± 2.9	11.7 ± 3.8	10.8 ± 3.2#
AVAD, ventricular ED, cm^2^	17.0 ± 3.4	19.6 ± 5.0*	18.7 ± 4.4
LV short-axis area, ventricular ES, cm^2^	15.6 ± 2.9	15.8 ± 2.9	14.7 ± 4.0
LA short-axis area, ventricular ES, cm^2^	21.7 ± 3.6	22.1 ± 4.1	21.0 ± 4.1
AVAD, ventricular ES, cm^2^	−6.0 ± 3.2	−6.3 ± 3.5	−6.3 ± 3.9

Values are means ± SD. AVAD, atrioventricular area difference; ED, end-diastole; ES, end-systole; LA, left atrial; LV, left ventricular.

* *P* < 0.05 for comparing rest to moderate exercise; #*P* < 0.05 for comparing rest to vigorous exercise; †*P* < 0.05 for comparing moderate to vigorous exercise.

### Atrioventricular Area Difference in ED

Results for AVAD at ventricular ED are presented in Fig. 3 and Table 3. No interaction was found between training status and exercise intensity for AVAD in ED. AVAD in ED increased when going from rest to moderate exercise for sedentary controls and remained unchanged when going from moderate to vigorous exercise. Similarly, AVAD in ED also increased for athletes when going from rest to moderate exercise, but not from moderate to vigorous exercise. When comparing the groups, there was no difference in AVAD in ED between sedentary controls and athletes for either rest or exercise.

### Atrioventricular Area Difference in ES

Results for AVAD at ventricular ES are also presented in Fig. 3 and Table 3. A significant interaction between training status and exercise intensity was seen for AVAD in ES (*P* = 0.008). For sedentary controls, AVAD in ES decreased when going from rest to moderate exercise but did not differ from moderate to vigorous exercise. In contrast, AVAD in ES for athletes did not differ between rest, moderate, or vigorous exercise. Furthermore, AVAD in ES differed between sedentary controls and athletes at rest (*P* = 0.007), but not for any of the exercise intensities.

### Intra- and Interobserver Variability

**Figure 5.**
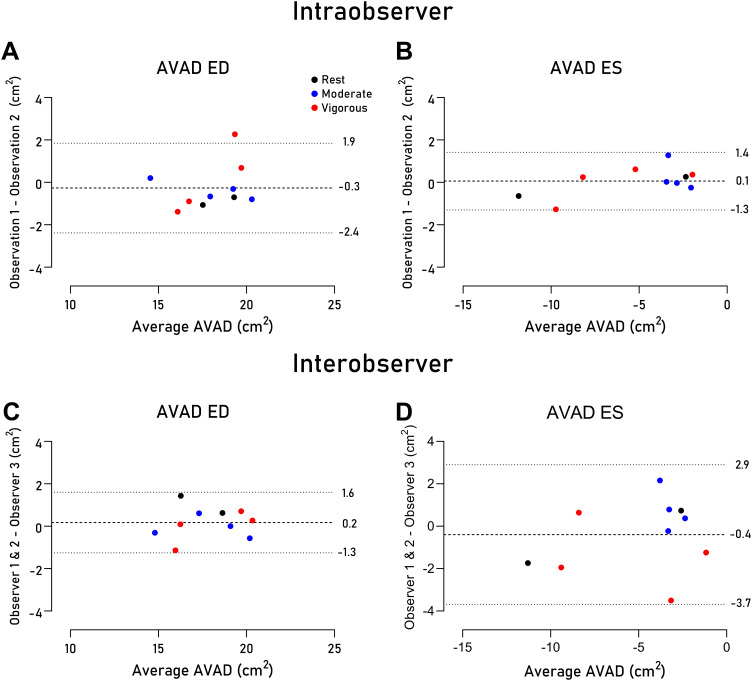
Bland–Altman plots presenting the intra- (*A* and *B*) and interobserver (*C* and *D*) variability of measurements of atrioventricular area difference (AVAD) in ventricular end-diastole (ED; *A* and *C*) and end-systole (ES; *B* and *D*) in 10 randomly selected datasets. Black dots indicate measurements from rest, blue from moderate exercise, and red from vigorous exercise. The dotted line denotes the bias, and the dashed lines the upper and lower limits of agreement (1.96 standard deviations).

Intra- and interobserver variability are presented in Fig. 5. Intraobserver variability was excellent with low bias for AVAD both in ED (−0.3 ± 1.1 cm^2^) and in ES (0.1 ± 0.7 cm^2^). Interobserver variability was also excellent with low bias for AVAD in ED (0.2 ± 0.7 cm^2^) and in ES (−0.4 ± 1.7 cm^2^).

## DISCUSSION

The current study set out to investigate changes in AVAD, and thus hydraulic force, during exercise in sedentary controls and athletes. The main finding showed that AVAD in ED is increased in both sedentary controls and athletes during moderate exercise compared with rest, with no interaction between training status and AVAD. This provides new mechanistic understanding of diastolic filling during exercise and indicates that the net hydraulic force aids LV diastolic filling to a greater extent during moderate exercise compared with rest, irrespective of training status.

### Atrioventricular Area Difference in ED during Exercise

Increased AVAD, and thus hydraulic force, in ED during exercise is likely caused by well-known physiological mechanisms occurring during exercise. These include increased venous return and subsequent increased ventricular filling, and increased atrial contraction, decreasing the volume of the atrium, and increasing the volume of the ventricle (16). Furthermore, atrioventricular plane displacement increases during exercise, mainly affecting atrial size (17, 18). These alterations in volumes affect the geometrical relationship between the atrium and ventricle that is advantageous for the hydraulic filling mechanism. Indeed, previous studies quantifying the hydraulic forces have shown hydraulic force to be an important mechanism contributing to diastolic filling in total, in the same order of magnitude as the elastic recoil of the myocardium at rest (9, 11).

Interestingly, AVAD in ED did not increase during vigorous exercise compared with rest for either group. This could result from physical exercise in this study being performed while in supine position, which significantly increases venous return already at rest. While venous return is expected to further increase from moderate to vigorous exercise, the venous return is likely already great enough from lying supine to not be a limiting factor for ventricular filling. Instead, AVAD not increasing could be explained by a shortened duration of diastole that occurs at high heart rates during physical exercise (19). If the diastolic phase is shortened enough to limit ventricular filling, the EDV, and consequently ventricular short-axis area, is reduced. Simultaneously, the atrial volume is not affected by the shortening of ventricular diastole, as atrial filling mainly occurs during ventricular systole (20).

### Atrioventricular Area Difference in ES during Exercise

The AVAD was always negative in ES, meaning that the direction of the net hydraulic force was toward the apex of the ventricle. This is in line with previous results in healthy research participants at rest (9, 10).

These results indicate hydraulic force to have a negative effect on diastolic filling at the very start of diastole. Furthermore, the interaction between AVAD in ES and training status could be interpreted as hydraulic force increasingly impairing filling in controls with increasing exercise intensity, but not in athletes. However, regardless of training status, this negative effect is likely counteracted by the elastic recoil, an important mechanism of diastolic function that primarily acts in the early rapid filling phase of diastole and further increases during exercise (21–26). This has been shown at rest in a study by Maksuti et al. (9) where the magnitude of the hydraulic force was small in comparison with that of the elastic recoil when measured at peak ventricular filling.

Thus, hydraulic force and elastic recoil could be considered two independent mechanisms of diastolic function that happen to synergize by elastic recoil primarily acting in early diastole, and hydraulic force primarily when the rapid filling phase is complete.

### Atrioventricular Area Difference over the Cardiac Cycle

Following the aforementioned reasoning, measuring how quickly into diastole AVAD switches from negative to positive, i.e., indicating when hydraulic force starts providing a boost to filling, is likely better for assessing the role of hydraulic force in early diastolic filling rather than only measuring AVAD in ES. Measuring AVAD over the cardiac cycle at rest has been done in previous studies and shown to differ when comparing healthy hearts to heart failure (9, 10). Unfortunately, time resolved examination of AVAD during exercise was not methodologically possible with the current data but would be of interest to include in future exercise CMR studies.

Another aspect of diastolic filling that needs to be considered in the context of hydraulic force is the atrial contraction. By measuring AVAD only in ED it could appear that hydraulic force is primarily a function of atrial contraction. However, the hydraulic force aiding diastolic filling is present also in the absence of atrial contraction, as shown in an experimental model by Maksuti et al. (9). The results of the experimental setup have later been confirmed in humans showing that the net hydraulic force directed toward the base starts augmenting ventricular filling already in the early rapid filling phase in healthy controls at rest, thus before atrial contraction begins (9, 10).

### Clinical Implications

Hydraulic force is a mechanism contributing to diastolic filling in healthy hearts (9). As it is affected by the geometrical relationship between the atrium and the ventricle, changes in these chambers may impair or improve diastolic filling, as shown during exercise in the current study. This diastolic mechanism has also been illustrated in previous studies of heart failure (10), heart transplantation (11), and congenital heart disease (27, 28). Furthermore, using echocardiography, Soundappan et al. (29) showed AVAD to provide additional prognostic value to conventional echocardiography parameters, with decreased AVAD in ED independently being associated with lower survival. Assessing AVAD during exercise could potentially provide additional diagnostic and prognostic data. For example, it may be a useful tool to unmask heart failure with preserved ejection fraction where exercise intolerance is a hallmark symptom but clinical implications of structural cardiac defects can be difficult to quantify with conventional methods.

### Limitations

The absence of invasive data regarding intracardiac pressure may be considered a limitation when discussing hydraulic force in this setting, as without these measurements we cannot calculate the magnitude of the hydraulic force. However, regardless of magnitude, AVAD provides the direction of the resulting net hydraulic force and thus whether it aids or impairs ventricular filling. As mentioned previously, AVAD has been shown to be an independent prognostic marker in a previous study (29). Future studies including intraventricular pressure measurements to enable quantification of the magnitude of the hydraulic force would be of interest to investigate if this could provide additional prognostic or diagnostic data.

One challenging and potentially limiting factor that should be addressed in this study is CMR image quality. The image quality declined when going from rest to moderate exercise, and even further to vigorous exercise. This was mainly due to increased bulk and cardiac motion caused by physical exercise and free breathing during exercise. Furthermore, image quality differs between the standard CMR cine sequence used for acquiring images at rest and the real-time CMR sequence used for exercise. Although the exercise images generally were of lower quality, the sequence and methods of analysis used for exercise in this study have previously been validated from our group and shown to provide low bias and high precision for LV mass and volumes when compared with rest in ED and ES (15).

One potential oversight in the study is the lack of a more detailed exercise protocol, as this could make it more difficult to reproduce the study. Although we used manual ramping until target heart rate was reached, the time required to reach, and the power output achieved, at the target heart rates were not noted down. From recollection, the time to ramp up was ∼1 min for reaching 60%, and 2 min for 80% of age-predicted maximum heart rate with a short rest between ramps. This was true for both controls and athletes, as resistance was manually ramped up and thus the rate of resistance increase was different for each participant.

### Conclusions

The atrioventricular area difference in ED increases during exercise. This creates a net hydraulic force that further augments diastolic filling during moderate exercise compared with rest, regardless of training status, providing new insights into the mechanism by which diastolic function increases during exercise. AVAD in ES was negative at rest and exercise for both groups. Although this indicates hydraulic force to impede ventricular filling at the very start of diastole, this effect is likely counteracted by elastic recoil.

## DATA AVAILABILITY

Data are available from the Cardiac MR Group in Lund, Lund University, Sweden for researchers who meet the criteria for access to confidential data and after additional consent from the research participants. For data access requests, please contact the Cardiac MR Group in Lund via email: cmr-lund@med.lu.se.

## GRANTS

This study was funded by The Swedish Research Council for Sport Science, Swedish Olympic Committee, Heart and Lung Foundation Grant 20220520, Södra Sjukvårdsregionen, and Medical Faculty at Lund University.

## DISCLAIMERS

The authors have no disclaimers to declare.

## DISCLOSURES

Einar Heiberg is the founder of software company Medviso AB, which develops the software Segment that was used in the image analysis in the current study. None of the other authors has any conflicts of interest, financial or otherwise, to disclose.

## AUTHOR CONTRIBUTIONS

H.A. and K.S.-E. conceived and designed research; B.Ö. and K.S.-E. performed experiments; J.E., B.Ö., and K.S.-E. analyzed data; J.E., H.A., and K.S.-E. interpreted results of experiments; J.E. prepared figures; J.E. drafted manuscript; J.E., B.Ö., E.H., H.A., and K.S.-E. edited and revised manuscript; J.E., B.Ö., E.H., H.A., and K.S.-E. approved final version of manuscript.
